# A systematic review and meta-analysis of Liuzijue in stable patients with chronic obstructive pulmonary disease

**DOI:** 10.1186/s12906-020-03104-1

**Published:** 2020-10-14

**Authors:** Lu Xiao, Hongxia Duan, Peijun Li, Weibing Wu, Chunlei Shan, Xiaodan Liu

**Affiliations:** 1 School of Rehabilitation Science, Shanghai University of Traditional Chinese Medicine, Shanghai, China; 2 Department of Rehabilitation, the Second Rehabilitation Hospital of Shanghai, Shanghai, China; 3 Department of Sports Medicine, Shanghai University of Sport, Shanghai, China; 4 Institute of Rehabilitation Medicine, Shanghai Academy of Traditional Chinese Medicine, Shanghai, China

**Keywords:** Chronic obstructive pulmonary disease, Liuzijue, Exercise

## Abstract

**Background:**

To investigate the effectiveness of Liuzijue exercise on chronic obstructive pulmonary disease (COPD) in the stable phase.

**Methods:**

We searched six electronic bibliographic databases (PubMed, EMBASE, The Cochrane Library, Web of Science, CNKI, and Wan Fang Data) from inception to August 2018. Randomized controlled trials (RCTs) were included if they evaluated the effect of Liuzijue exercise on stable COPD. Cochrane Collaboration risk-of-bias tool (Cochrane Handbook 5.1.0) was used to assess the risk of bias of included RCTs. Meta-analysis was performed using the Review Manager software (RevMan V.5.3.5) provided by the Cochrane Collaboration. Outcomes assessed included dyspnea, exercise capacity, lung function, and quality of life.

**Results:**

Fourteen RCTs involving 920 stable COPD patients were included in this systematic review and meta-analysis. The control groups received usual care. The average number of training sessions per participant was 9.3 per week, and the average length of these training sessions was 31.6 min per week. Training duration varied from 3 to 12 months. Meta-analysis results showed that Liuzijue exercise can effectively improve patients’ Modified Medical Research Council Dyspnea Scale scores (MD = − 0.73, 95% CI: − 1.13 to − 0.33, *P* < 0.05), 6MWD (MD = 17.78, 95% CI: 7.97 to 27.58, *P* < 0.05), forced expiratory volume in one second (FEV_1_) (MD = 0.23, 95% CI: 0.07 to 0.38, *P* < 0.05), the percentage of predicted values of FEV_1_ (FEV_1_%pred) (MD = 7.59, 95% CI: 2.92 to 12.26, *P* < 0.05), FEV_1_/FVC (Forced vital capacity) ratio (MD = 6.81, 95% CI: 3.22 to 10.40, *P* < 0.05), Quality of life: St. George’s Respiratory Questionnaire total score (MD = − 9.85, 95%CI: − 13.13 to − 6.56, *P* < 0.05), and Chronic Obstructive Pulmonary Disease Assessment Test score (MD = − 2.29, 95%CI: − 3.27, − 1.30, *P <* 0.05).

**Conclusion:**

Evidence from meta-analysis suggested that Liuzijue exercise could improve dyspnea, exercise endurance, lung function, and quality of life for stable COPD patients. However, owing to the methodological bias and the placebo effect of Liuzijue exercise, there is a need for further research to confirm these findings.

**Trial registration:**

PROSPERO (ID: CRD42019130973).

## Background

Chronic obstructive pulmonary disease (COPD) is a common chronic pulmonary disease with high morbidity and mortality. The characteristics of COPD patients are persistent airflow restriction and respiratory symptoms resulting from airway and/or alveoli abnormalities caused by long-term exposure to noxious particles or gases [1]. A *Lancet* study showed that the prevalence of COPD has increased from 8.2 to 13.7% among Chinese people over the age of 40 [2, 3]. In 2016, there were 251 million cases of COPD and about 3 million deaths caused by COPD worldwide [4]; the direct medical cost ranged from 72 to 3563 USD per capita per year [5]. COPD is the fourth leading cause of death in the world at present, and the World Health Organization has predicted that it will become the third leading cause by 2030 [6]. Owing to the high morbidity, mortality, and massive economic burdens it presents society, increasing numbers of researchers are paying close attention to the prevention and treatment of COPD.

Lung function tests are essential for COPD diagnosis. Studies have shown that worsening COPD severity is associated with higher mortality and hospitalization [7]. A low FEV_1_ (forced expiratory volume in one second)/FVC (Forced vital capacity) ratio is also related to higher mortality [8]. In addition to decrease lung function and long-lasting respiratory symptoms, COPD patients may have skeletal muscle dysfunction, such as decreased muscle strength [9, 10] and exercise endurance [1]. These symptoms can lead to lower exercise capacity and a worse quality of life. An exercise capacity assessment includes assessing muscle strength and exercise endurance. The six-minute walking test is an efficient tool for assessing exercise endurance, which is closely related to many functional outcomes for COPD patients [11, 12]. St. George’s Respiratory Questionnaire (SGRQ) and the Chronic Obstructive Pulmonary Disease Assessment Test (CAT) are common measurements of quality of life. The SGRQ and CAT scores are significantly correlated and both with high reliability and validity [13–15]. An improvement in both scores is significantly correlated with improvements in Modified Medical Research Council Dyspnea Scale (mMRC) score, lung function, and risk of COPD exacerbation [16–21]. However, the correlation between CAT score and pulmonary function, dyspnea, exercise performance, and psychological factors is lower than its correlation with total SGRQ score [14, 19]. The relevance between total SGRQ score and COPD disease severity is higher [22].

The *Global Initiative for Chronic Obstructive Lung Disease* has identified pulmonary rehabilitation as an essential prevention and control measure for COPD. As the cornerstone of pulmonary rehabilitation, exercise training showed significant improvement for dyspnea, quality of life, exercise endurance, and rate of hospital re-admissions in COPD patients [1]. Traditional Chinese exercises are a type of exercise training that is widely used in pulmonary rehabilitation programs for clinically stable COPD patients. Many clinical studies suggest that traditional Chinese exercise can improve the health of COPD patients [23–25]. Liuzijue is a moderate-low intensity traditional Chinese exercise. Breathing is an important component of Liuzijue; when coordinated with simple body movements, breathing can strengthen the body and improve the bodily function of Liuzijue practitioners. Liuzijue exercises include six pronunciations: ‘XU’, ‘HE’, ‘HU’, ‘SI’, ‘CHUI’, and ‘XI’. Different pronunciations will produce different sound frequencies and resonate with different parts of the body, which is beneficial for health [26]. It is necessary to adopt long and steady abdominal breathing and pursed-lip breathing when practicing Liuzijue. The movements of Liuzijue contain stretching, flexion, and extension of upper limbs, rotation of the body, expansion of the chest, and contraction and relaxation of the abdomen [27, 28]. Upper limb exercise can improve the lung function, dyspnea, and quality of life of COPD patients [29, 30]. The vocal organs exert different forces during different pronunciations, and when coordinated with different body movements, can exercise the major and accessory respiratory muscles [31], improve dyspnea, regulate lung function, and increase exercise capacity, eventually improving the patient’s quality of life [31, 32]. Based on the characteristics of breathing methods and body movements of Liuzijue, we considered that it may be suitable for the rehabilitation needs of COPD patients.

At present, many clinical studies suggest that traditional Chinese exercise can improve the condition of COPD patients. Many meta-analyses have already [33–36] investigated the effects of multiple traditional Chinese exercises for COPD patients, but meta-analysis about the effects of Liuzijue in COPD patients is still lacking. Therefore, we exclusively searched for and collated relevant Chinese and English studies focusing on Liuzijue exercise. Subsequently, we conducted a systematic review and meta-analysis to clarify whether practicing Liuzijue exercise is beneficial to respiratory symptoms, lung function, exercise capacity, and quality of life in patients with stable COPD.

## Methods

Inclusion and exclusion criteria, as well as analytical methods, were registered in the PROSPERO (http://www.crd.york.ac.uk/PROSPERO) database (PROSPERO number, CRD42019130973).

### Selection criteria

Eligibility criteria: (1) Study type: RCTs published in Chinese and English. (2) Subjects: patients diagnosed with stable COPD. (3) Interventions: the control group was given routine drug treatment and health guidance; the intervention group received Liuzijue or simplified Liuzijue exercise. (4) Outcomes: A. Dyspnea: Medical Research Council Dyspnea Scale (MRC) or mMRC; B. Exercise capacity: maximal inspiratory pressure (MIP), maximal expiratory pressure (MEP), limb muscle strength, six-minute walking distance (6MWD), 30 Second Sit-to-Stand Test (30s SST); C. Lung Function: FEV_1_, the percentage of predicted values of FEV_1_ (FEV_1_%pred), and FEV_1_/FVC ratio (FEV_1_/ FVC%); D. Quality of life: SGRQ, CAT.

Exclusion criteria: (1) Inconsistent with our intervention measures; (2) outcomes outside the specified range; (3) duplicated data; (4) full-text was not available; (5) not RCT or with inappropriate random method.

### Data sources and search strategy

We searched six databases (PubMed, EMBASE, The Cochrane Library, Web of Science, CNKI, and Wan Fang Data) from inception to August 2018. The search was conducted with the following keywords in Chinese and English: Liuzijue (Qigong OR Qi gong OR chi gong OR chi kung OR traditional Chinese exercises) and Chronic Obstructive Pulmonary Disease (Chronic Airflow Obstruction OR COPD OR Chronic Obstructive Airway Disease OR Chronic Obstructive Lung Disease).

### Study selection and data extraction

Two researchers (LX and HXD) independently extracted data from the selected studies based on the predetermined inclusion and exclusion criteria. Each disagreement was solved with the help of a third reviewer (XDL) when necessary. The following information was extracted from each included study: first author, the year of publication, sample size, average age, proportion of male and female patients, disease severity level, intervention measures, intervention plan for control group and Liuzijue group, and related outcomes (mMRC/MRC score, MIP, MEP, limb muscle strength, 6MWD, FEV_1_, FEV_1_%pred, FEV_1_ / FVC%, SGRQ, and CAT score).

### Assessment of risk of bias

We used the Cochrane Collaboration risk-of-bias tool (Cochrane Handbook 5.1.0) to assess risk of bias of included RCTs. Potential sources of bias include random sequence generation, allocation concealment, blinding of participants and staff, blinding of outcome assessors, incomplete outcome data, selective reporting, and other biases. Each trial received a study level score of low, high, or unclear risk of bias for each domain. Two authors (LX and HXD) independently conducted this assessment, and discrepancies were resolved through discussion with a third person (PJL).

### Data synthesis

Meta-analysis was performed using the Review Manager statistical software (RevMan V.5.3.5) provided by the Cochrane collaboration. We analyzed the statistics by the mean difference (MD) and 95% confidence interval (95% CI). A *P* value of < 0.05 was regarded as statistically significant. The heterogeneity of included studies was assessed using I^2^ statistics. When the I^2^ statistic value was less than 50%, the random effects model was used; otherwise, the fixed effects model was used to measure outcomes with heterogeneity.

## Results

### Search results

**Fig. 1:**
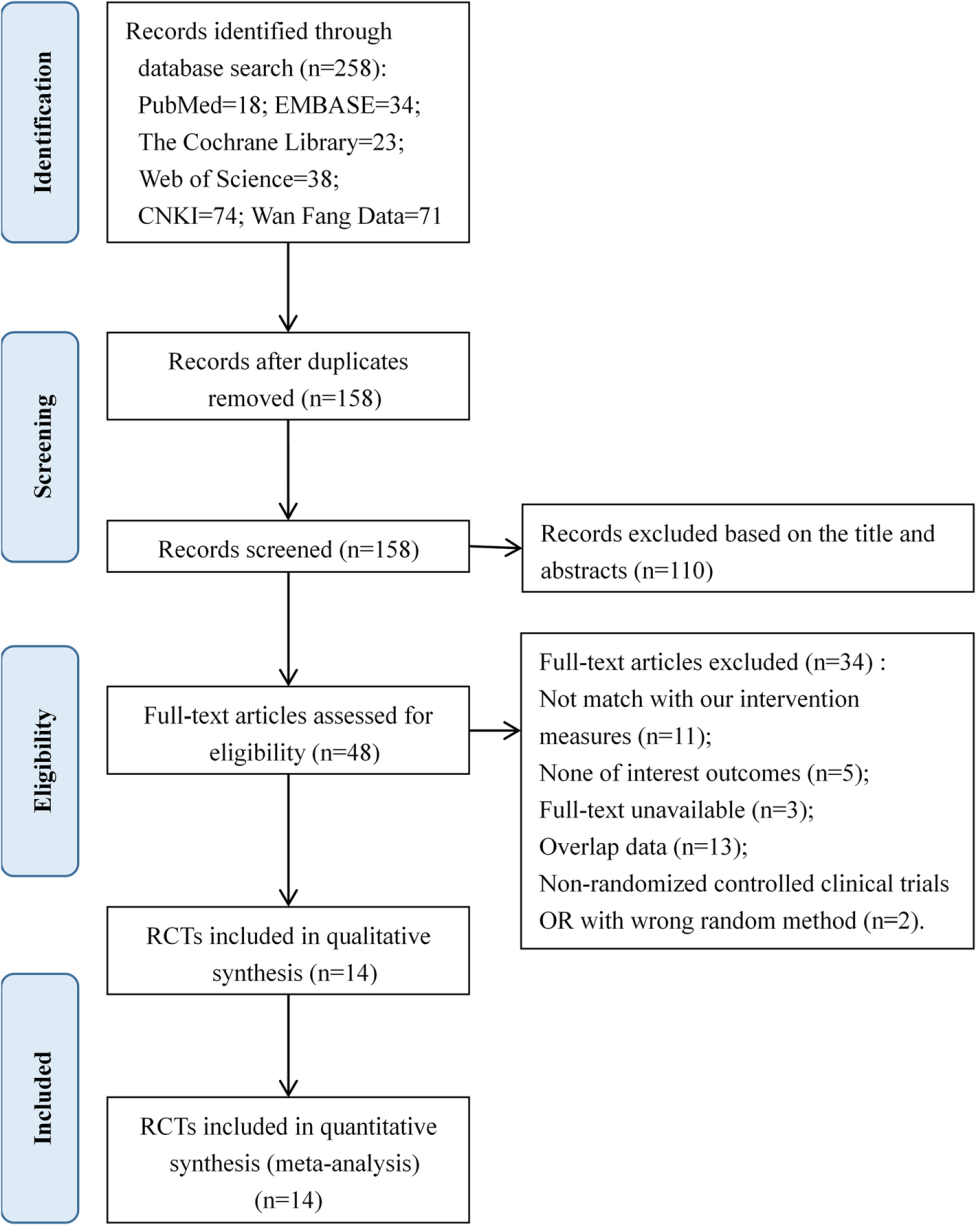
Selection process for included studies

A total of 258 records (145 in Chinese and 113 in English) were initially identified from the six databases. After excluding 100 duplications, 158 studies were chosen for further evaluation. Through screening the titles and abstracts, 110 studies were excluded. We searched for the full text of the remaining 48 studies, and 14 studies were ultimately included while 34 were excluded (inconsistent with intervention measures = 11, outcomes outside the specified range = 5, duplicate data = 13, full text was not available = 3, not RCT or with inappropriate random method = 2). Finally, 14 studies were included for our meta-analysis (Fig. 1).

### Characteristics of included studies

Fourteen RCTs [32, 37–49] published from 2009 to 2018 (of which 12 RCTs were published in Chinese [37–48] and two were published in English [32, 49]) were included. A total of 920 patients (control group = 463, Liuzijue group = 457) were pooled from all the included trials into our final systematic review and meta-analysis. These patients ranged from 39 to 82.26 years old with a COPD severity stage ranging from mild to very severe. The control group received routine drug therapy and health guidance. The average number of training sessions per participant was 9.3 per week and the average length of these training sessions was 31.6 min per week. Training duration ranged from 3 to 12 months. The characteristics of the included studies are shown in Table S1.

### Risk of bias

**Fig. 2:**
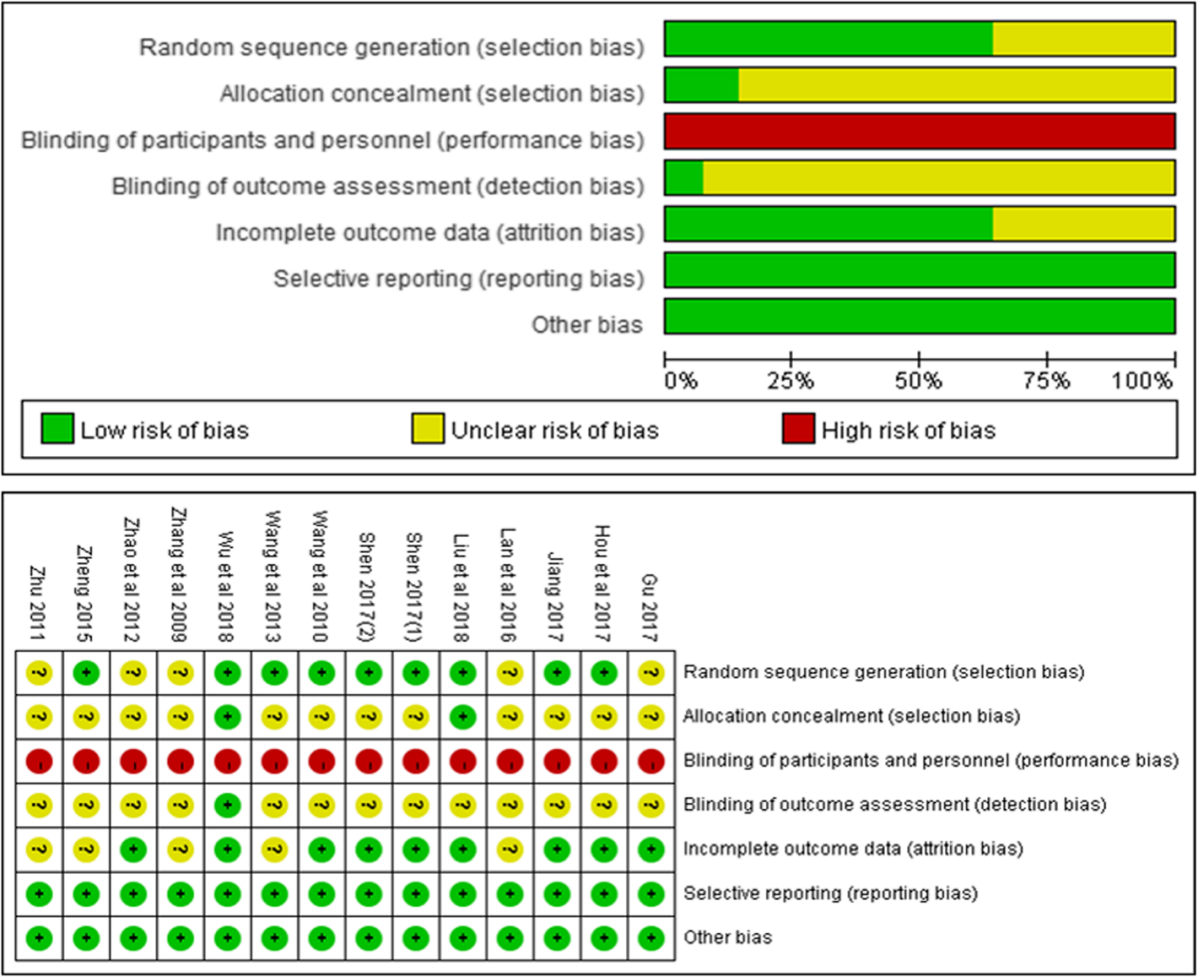
Assessment of Risk of bias

Details of the risk of bias assessment for the 14 included RCTs are shown in Fig. 2. We used the Cochrane tool (Cochrane Handbook 5.1.0) to assess the risk of bias. The method of random sequence generation, which was either by random number table or computer random number generator, was mentioned in nine studies [32, 38, 41, 42, 44–46, 48, 49], while other studies did not mention the random method. Two studies [32, 49] reported information about allocation concealment. As the Liuzijue exercises require active engagement, blinding of participants and staff was impossible. Only one study reported the blinding of the assessor [49]. Nine studies [32, 38, 39, 44–49] clearly described the reasons and the number of exclusion cases. All studies [32, 37–49] have reported all the predefined outcomes.

### Meta-analysis results

#### Effect of Liuzijue on dyspnea

**Fig. 3:**
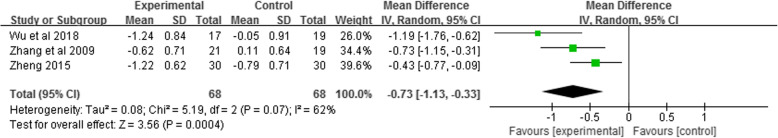
Meta-analysis of the effect of Liuzijue on MRC (or mMRC). Abbreviations: MRC, Medical Research Council Dyspnea Scale; mMRC, Modified Medical Research Council Dyspnea Scale

MRC (or mMRC) scores were investigated in three included studies [37, 42, 49] (136 COPD patients). Compared with the control group, improvement in MRC (or mMRC) scores in the Liuzijue group was more significant (MD = − 0.73, 95% CI: − 1.13 to − 0.33, I^2^ = 62%, *P* < 0.05, Fig. 3).

#### Effect of Liuzijue on respiratory muscle strength

MIP and MEP were reported in one study [32]. In the study [32], compared with the control group, the respiratory muscle strength (MIP, MEP) of patients in the Liuzijue group showed significant improvement (*P* < 0.01).

#### Effect of Liuzijue on limb muscle strength

Of the 14 studies, only two studies [32, 49] mentioned the assessment of limb muscle strength. A study by Wu et al. [49] reported the efficiency of Liuzijue exercise on patients’ handgrip strength. Compared with 6 months prior, the handgrip strength of COPD patients in the Liuzijue group rose an average of 4% (*P* = 0.03). The results were statistically significant, but there was no significance compared with the control group. Liu et al. [32] reported the effect of Liuzijue on the muscles around the elbow and knee joints. Total work (TW) of the elbow extensor, elbow flexor, and knee extensor, peak torque (PT), and the ratio of peak torque to body weight (PT/BW) of the knee extensor were significantly improved in Liuzijue group after 3 months. The muscle endurance ratio (ER) of the elbow extensor and the TW of the elbow flexor and knee flexor in the Liuzijue group increased significantly compared with the control group (*P* < 0.05).

#### Effect of Liuzijue on exercise endurance

**Fig. 4:**
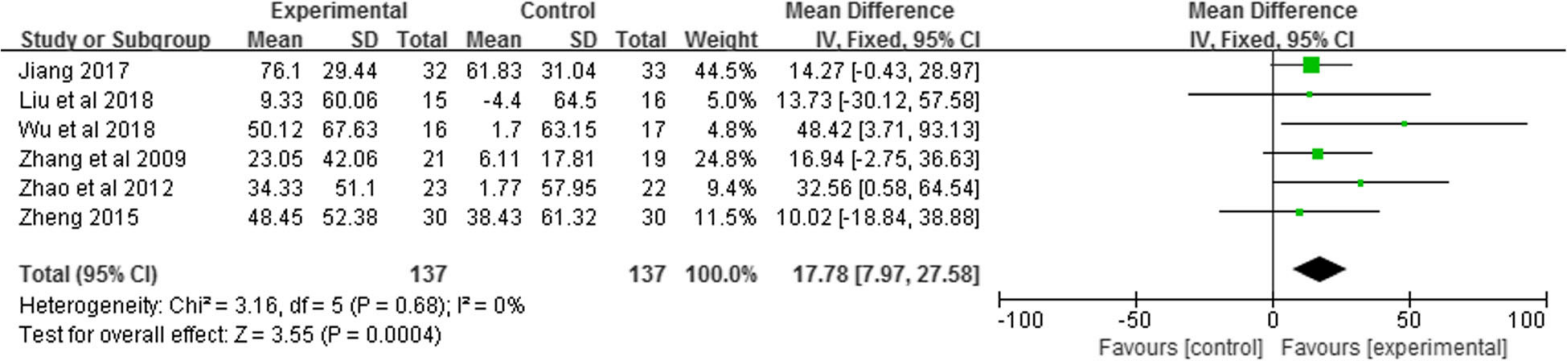
Meta-analysis of the effect of Liuzijue on 6MWD. Abbreviations: 6MWD, 6 min walking distance

Six studies [32, 37, 39, 42, 44, 49] with a total of 274 patients were included in the meta-analysis of 6MWD. The forest plot showed that the 6MWD improvement in the Liuzijue group was significantly greater than that in the control group (MD = 17.78, 95% CI: 7.97 to 27.58, I^2^ = 0%, *P <* 0.05, Fig. 4). In the study by Wu et al. [49], 30s SST was also conducted to evaluate patients’ exercise endurance. In this study, 30 s SST times of the Liuzijue group increased significantly, but there was no significant difference between the two groups.

#### Effect of Liuzijue on lung function

**Fig. 5:**
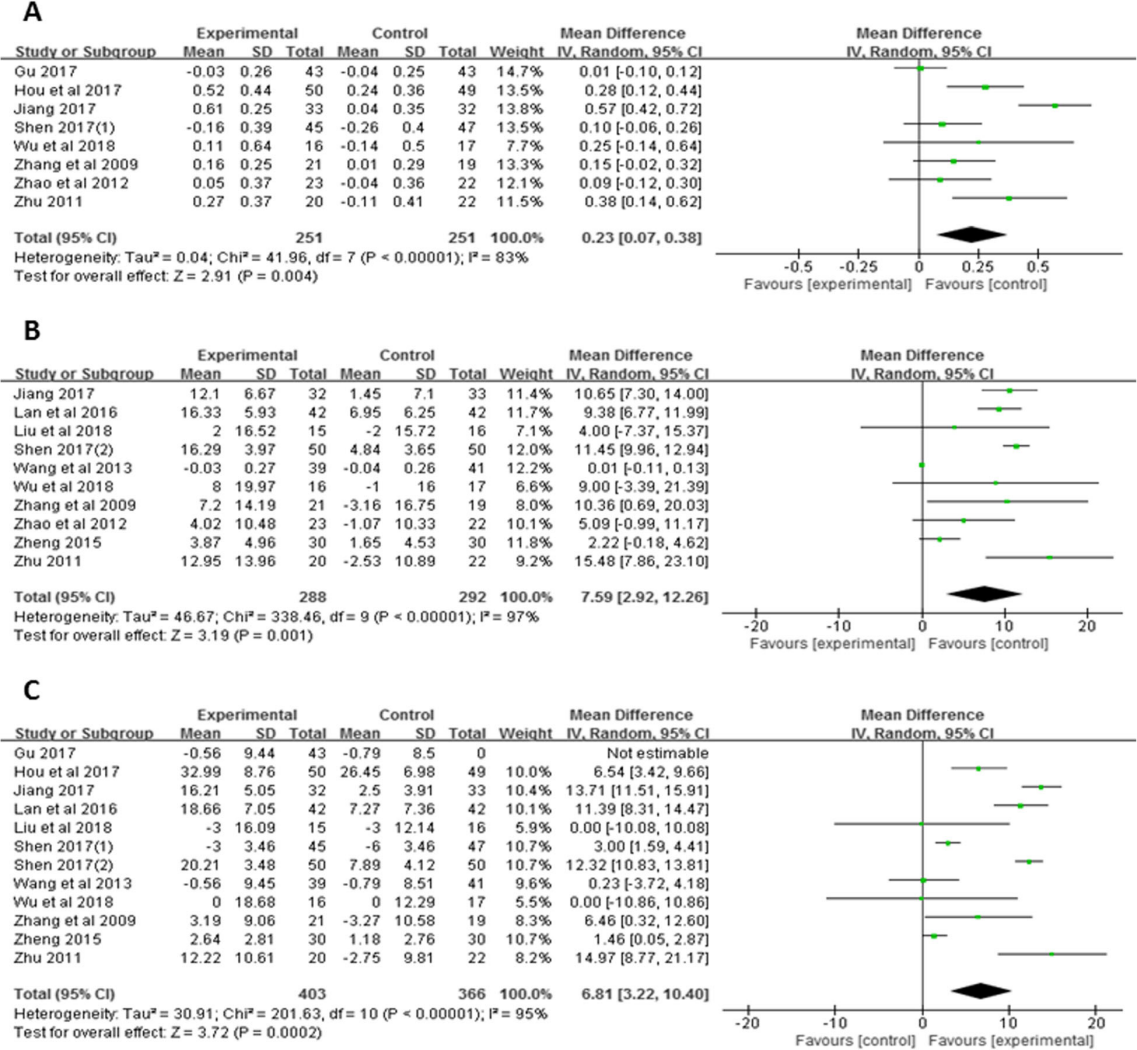
Meta-analysis of the effect of Liuzijue on lung function: **a** FEV_1_; **b** FEV_1_%pred; **c** FEV_1_/FVC%. Abbreviations: FEV_1_, forced expiratory volume in one second; FEV_1_%pred, the percentage of predicted values of FEV_1_; FEV_1_/ FVC%, FEV1/FVC ratio

Eight studies [37, 39, 40, 44, 45, 47–49] with a total of 502 patients were eligible for FEV_1_ analysis. Compared with the control group, Liuzijue exercise had a significant effect on FEV_1_ improvement in COPD patients (MD = 0.23, 95% CI: 0.07 to 0.38, I^2^ = 83%, *P* < 0.05, Fig. 5a).

Ten studies [32, 37, 39–44, 46, 49] with a total of 580 patients were included for the FEV_1_%pred analysis. The results showed that the FEV_1_%pred of COPD patients in the Liuzijue group was significantly higher than that in the control group (MD = 7.59, 95% CI: 2.92 to 12.26, I^2^ = 97%, *P <* 0.05, Fig. 5b).

Twelve studies [32, 37, 40–49] with a total of 769 patients were eligible for the analysis of FEV_1_/FVC_%_. The improvement of FEV_1_/FVC% in the Liuzijue group was significantly higher than that in the control group (MD = 6.81, 95% CI: 3.22 to 10.40, I^2^ = 95%, *P <* 0.05, Fig. 5c).

#### Effect of Liuzijue on quality of life

Among the 14 studies, a total of nine studies assessed quality of life. Five of the nine studies [38, 40, 42, 48, 49] used SGRQ, while four studies [43–46] used CAT.

**Fig. 6:**
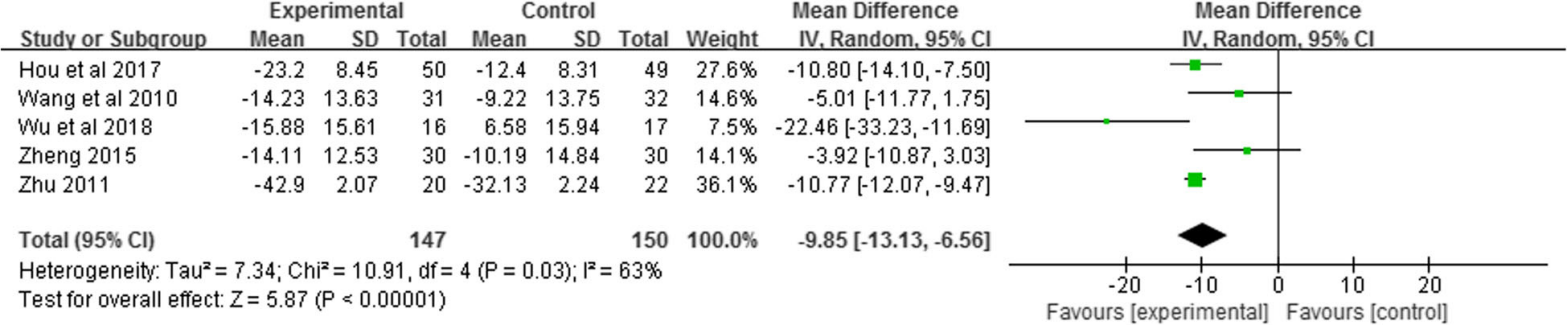
Meta-analysis of the effect of Liuzijue on SGRQ total scores. Abbreviations: SGRQ, St. George’s Respiratory Questionnaire

Five studies [38, 40, 42, 48, 49] (297 COPD patients) that reported SGRQ scores were included in the meta-analysis. This suggests that the total SGRQ scores of patients in the Liuzijue group decreased more significantly (MD = − 9.85, 95% CI: − 13.13 to − 6.56, I^2^ = 63%, *P* < 0.05, Fig. 6) than those of the control group. Additionally, four [38, 40, 42, 49] (198 COPD patients) of the five studies reported three domains (symptom, activity, and influence) of SGRQ. The results showed that the differences in SGRQ score for symptoms and activity between the two groups were significant (*P <* 0.05, Fig. S1).

**Fig. 7:**
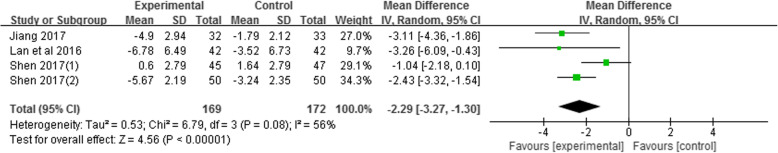
Meta-analysis of the effect of Liuzijue on CAT scores. Abbreviations: CAT, Chronic Obstructive Pulmonary Disease Assessment Test

Four studies [43–46] with a total of 341 COPD patients were included in the meta-analysis of CAT scores. The results showed that the reduction of CAT scores in the Liuzijue group was more significant than that in the control group (MD = − 2.29, 95% CI: − 3.27, − 1.30, I^2^ = 56%, *P <* 0.05, Fig. 7).

### Adverse events

A total of 10 participants across six studies reported adverse events [39, 41, 45, 47–49], while the other studies did not report (Table S2). In the control group, one participant experienced severe pneumonia and respiratory failure [39] and two developed symptoms of herpes zoster [41, 47]. In the Liuzijue group, two participants died [41, 47], one reported exacerbation of COPD [49], and one was hospitalized due to other medical diseases [45]. There were three participants who experienced other serious diseases [48], but the grouping was unknown. However, there is no indication that these adverse events were caused by the research methods of the six studies.

## Discussion

### Dyspnea

The minimal clinical important difference (MCID) of MRC in patients with COPD is one unit [52]. There were significant clinical differences between two groups in two [42, 49] of the three studies. In these two studies, patients practiced Liuzijue at least 5 days (200 min) per week, and the duration was 6 months. According to the results, we can see that Liuzijue exercise was superior to routine therapy in improving patients’ dyspnea. The possible reasons may be as follows: the different forces of the lips, teeth, tongue, and larynx when pronouncing the six different sounds may have exercised patients’ respiratory muscles. The main breathing methods of Liuzijue are abdominal breathing and pursed-lip breathing. Pursed-lip breathing can improve dynamic lung hyperinflation, slow down breathing rate, and reduce airway resistance and airway stricture when exhaling, so as to improve patients’ dyspnea [53–55]. Moreover, pronunciation when practicing Liuzijue can lengthen the breathing time of COPD patients, which may improve gas retention.

### Exercise capacity

The results of a study by Liu et al. [32] revealed that Liuzijue exercise could significantly improve a patient’s respiratory muscle strength (MIP, MEP). However, due to the lack of relevant studies, the improvement of respiratory muscle strength in COPD patients practicing Liuzijue still cannot be determined.

Wu et al. [49] measured the handgrip strength of COPD patients and found that the improvement of handgrip strength in the Liuzijue group improved after 3 months. However, clinical significance (15%) was not achieved in this study. Liu et al. [32] assessed isokinetic muscle strength of elbow and knee joint muscle groups, and the comparison parameters included the PT, PT/BW, TW, and ER of two groups. The results revealed that elbow extensor TW achieved the MCID (15%). Handgrip strength and isokinetic muscle strength parameters were only reported by one study. It cannot yet be determined whether Liuzijue can improve limb muscle strength in COPD patients.

6MWD improved significantly in the Liuzijue group of 5/6 studies, and 4/5 studies [39, 42, 44, 49] achieved the minimal clinical significance indicator of 25 m [50]. Patients practiced Liuzijue at least 5 days (200 min) a week, and training duration was 3 months (one study) or 6 months (three studies) in these four studies. Heterogeneity between the studies was not apparent, which means the results were credible. These findings indicate that Liuzijue can significantly improve the exercise endurance of COPD patients. In a study by Wu et al. [49], 30s SST was applied to evaluate the exercise ability of patients, but improvement was not notable between the two groups. Owing to the low sample size of this study and the fact that this indicator was not used in other studies, the improvement in 30s SST times is still uncertain.

### Lung function

FEV_1_ improved significantly in the Liuzijue group of 4/8 studies, and four studies [37, 40, 44, 48] achieved the MCID of 0.1 L [51]. Patients practiced at least 30 min a day, 7 days a week, and practice duration was 3 months (three studies) or 6 months (one study) in these four studies. The meta-analysis results suggest that Liuzijue exercise can significantly improve the lung function (FEV_1_, FEV_1_%pred, FEV_1_/FVC%) of patients. However, because the heterogeneity between studies is obvious, the conclusions should be treated cautiously. We attempted to perform subgroup analysis for pulmonary function indicators, but the heterogeneity between the studies has not been eliminated. It may be caused by the variations in age, sex ratio, and disease severity among the studies.

### Quality of life

The MCIDs of SGRQ and CAT in patients with COPD were − 7.43 units and − 2.54 units, respectively [56]. All studies reported SGRQ [38, 40, 42, 48, 49] and three studies reported CAT [43, 44, 46], showing significant clinical differences between the two groups. Liuzijue practice frequency varied from 20 to 60 min a day, and at least 5 days (140 min) a week. Practice duration was 3 months (four studies) or 6 months (four studies) in these eight studies. Meta-analysis results showed that compared with the control group, Liuzijue exercise had a significant effect on the improvement of quality of life (CAT score, SGRQ total score, and symptom and activity scores of SGRQ) in COPD patients. However, there was significant heterogeneity among the studies. Differing disease severity in the participants and varying intervention times may be the possible reasons for this heterogeneity. Therefore, the identification of the improvement effect of Liuzijue exercise on patients’ quality of life requires further research.

## Limitations

Several limitations of the present study should be noted. First, as the Liuzijue exercises require active engagement, blinding of participants and staff was impossible. Additionally, a number of studies have not reported a blind method of independent outcome assessment, which could minimize the expectation bias of outcomes. This may result in an overestimation of the effect of Liuzijue on COPD patients. Second, because there was a lack of long-term follow-up studies, we cannot ensure the long-term effect of Liuzijue on COPD patients. Finally, our review only searched for relevant studies published in Chinese and English, and was unable to include all relevant research, which may lead to a results bias.

## Conclusion

Our study suggested that Liuzijue could effectively improve dyspnea, exercise capacity, lung function, and quality of life for COPD patients. For those COPD patients with apparent dyspnea, Liuzijue training frequency should reach at least 150 min per week, 5 days a week. However, there is only modest information about the effect of Liuzijue on stable COPD, which primarily comes from small, inadequate studies with potential risk of bias. That bias may increase the obvious benefits of Liuzijue, which means the evidence of benefit from Liuzijue was of low quality. Also, the likelihood is high that the effect will be substantially different from the estimate in this systematic review.

In addition, we have the following suggestions for future research. First, the clinical trial methodologies of many studies, such as random sequence generation, allocation concealment, and blinded independent outcome assessment, are not rigorous enough. The safety of Liuzijue has not been reported by some studies. We hope that future research will be further strengthened in methodology. Second, the intervention duration in current studies is mostly either three or 6 months. Large-sample, high-quality RCTs are needed in the future to investigate the long-term effect of Liuzijue on COPD patients. Third, most existing studies only assessed exercise endurance; there is a lack of research on the effect of Liuzijue on patients’ muscle strength. More studies investigating the effect of Liuzijue on patients’ respiratory muscle and limb muscle strength are needed. Finally, future rehabilitation programs must be more personalized according to the severity of COPD patients.

## Supplementary information

**Additional file 1: Table S1** Characteristics of included studies. CG = Control Group; LG = Liuzijue Group; “ - ”=Not reported in the study; MRC = Medical Research Council Dyspnea Scale; mMRC = Modified Medical Research Council Dyspnea Scale; MIP = Maximal Inspiratory Pressure; MEP = Maximal Expiratory Pressure; 6MWD = six-minutes walking distance; 30s SST =30s Sit-to-Stand Test; FEV1 = Forced expiratory volume in one second; FEV1%pred = the percentage of predicted values of FEV1; FVC = Forced vital capacity; SGRQ = St. George’s respiratory questionnaire; CAT = Chronic Obstructive Pulmonary Disease Assessment Test.

**Additional file 2: Table S2** Adverse Events.

**Additional file 3: Fig. S1** Meta-analysis of the effect of Liuzijue on SGRQ score: (A) Symptom; (B) Activity; (C) Influence**.** Abbreviations: SGRQ, St. George’s Respiratory Questionnaire.

## Data Availability

The datasets used and analyzed during the current study are available from the corresponding author upon reasonable request.

## Abbreviations

30s SST: 30 s Sit - to - Stand Test
6MWD: 6 min walking distance
BW: Body weight
CAT: Chronic Obstructive Pulmonary Disease Assessment Test
CG: Control group
CI: Confidence Interval
CNKI: China Network Knowledge Infrastructure
COPD: Chronic Obstructive Pulmonary Disease
ER: Muscle endurance ratio
FEV_1_: Forced expiratory volume in one second
FEV_1_%pred: Percentage of predicted values of FEV_1_
FVC: Forced vital capacity
LG: Liuzijue group
MD: Mean difference
MEP: Maximal expiratory pressure
MIP: Maximal inspiratory pressure
mMRC: Modified Medical Research Council Dyspnea Scale
MRC: Medical Research Council Dyspnea Scale
PT: Peak torque
RCTs: Randomized controlled trials
SGRQ: St. George’s Respiratory Questionnaire
TW: Total work
