# Supplementary figures and images for: A systematic review and meta-analysis of Liuzijue in stable patients with chronic obstructive pulmonary disease
Source: BMC Complement Med Ther. 2020 Oct 14;20:308. doi: 10.1186/s12906-020-03104-1 (PMC7557061; doi:10.1186/s12906-020-03104-1)

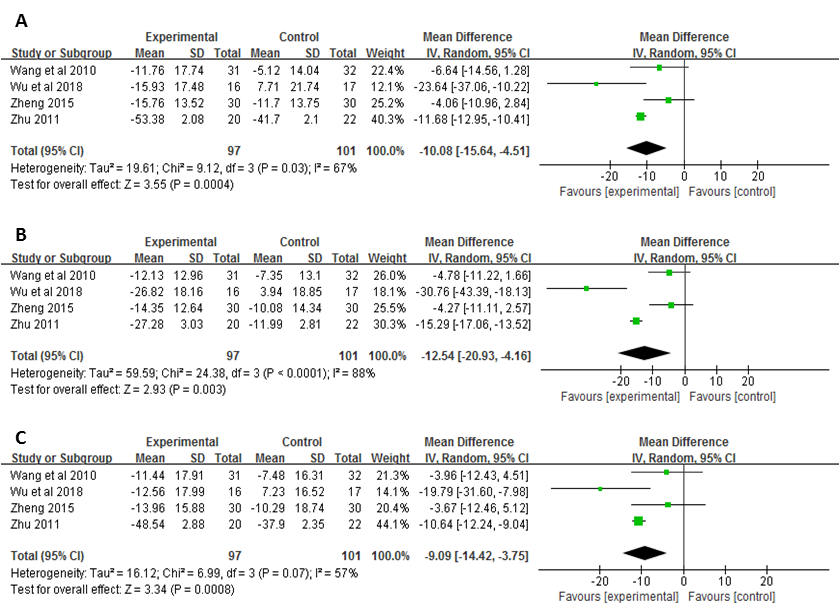

Supplement: Supplementary file 3 — Additional file 3: Fig. S1 Meta-analysis of the effect of Liuzijue on SGRQ score: (A) Symptom; (B) Activity; (C) Influence. Abbreviations: SGRQ, St. George’s Respiratory Questionnaire. [file 12906_2020_3104_MOESM3_ESM.png]
